# Reinforcement Learning to Optimize Ventilator Settings for Patients on Invasive Mechanical Ventilation: Retrospective Study

**DOI:** 10.2196/44494

**Published:** 2024-10-16

**Authors:** Siqi Liu, Qianyi Xu, Zhuoyang Xu, Zhuo Liu, Xingzhi Sun, Guotong Xie, Mengling Feng, Kay Choong See

**Affiliations:** 1 National University of Singapore Graduate School for Integrative Science and Engineering National University of Singapore Singapore Singapore; 2 Saw Swee Hock School of Public Health National University of Singapore Singapore Singapore; 3 Ping An Healthcare Technology Beijing China; 4 Institute of Data Science National University of Singapore Singapore Singapore; 5 Division of Respiratory and Critical Care Medicine Department of Medicine National University Hospital Singapore Singapore

**Keywords:** mechanical ventilation, reinforcement learning, artificial intelligence, validation study, critical care, treatment, intensive care unit, critically ill, patient, monitoring, database, mortality rate, decision support, support tool, survival, prognosis, respiratory support

## Abstract

**Background:**

One of the significant changes in intensive care medicine over the past 2 decades is the acknowledgment that improper mechanical ventilation settings substantially contribute to pulmonary injury in critically ill patients. Artificial intelligence (AI) solutions can optimize mechanical ventilation settings in intensive care units (ICUs) and improve patient outcomes. Specifically, machine learning algorithms can be trained on large datasets of patient information and mechanical ventilation settings. These algorithms can then predict patient responses to different ventilation strategies and suggest personalized ventilation settings for individual patients.

**Objective:**

In this study, we aimed to design and evaluate an AI solution that could tailor an optimal ventilator strategy for each critically ill patient who requires mechanical ventilation.

**Methods:**

We proposed a reinforcement learning–based AI solution using observational data from multiple ICUs in the United States. The primary outcome was hospital mortality. Secondary outcomes were the proportion of optimal oxygen saturation and the proportion of optimal mean arterial blood pressure. We trained our AI agent to recommend low, medium, and high levels of 3 ventilator settings—positive end-expiratory pressure, fraction of inspired oxygen, and ideal body weight–adjusted tidal volume—according to patients’ health conditions. We defined a policy as rules guiding ventilator setting changes given specific clinical scenarios. Off-policy evaluation metrics were applied to evaluate the AI policy.

**Results:**

We studied 21,595 and 5105 patients’ ICU stays from the e-Intensive Care Unit Collaborative Research (eICU) and Medical Information Mart for Intensive Care IV (MIMIC-IV) databases, respectively. Using the learned AI policy, we estimated the hospital mortality rate (eICU 12.1%, SD 3.1%; MIMIC-IV 29.1%, SD 0.9%), the proportion of optimal oxygen saturation (eICU 58.7%, SD 4.7%; MIMIC-IV 49%, SD 1%), and the proportion of optimal mean arterial blood pressure (eICU 31.1%, SD 4.5%; MIMIC-IV 41.2%, SD 1%). Based on multiple quantitative and qualitative evaluation metrics, our proposed AI solution outperformed observed clinical practice.

**Conclusions:**

Our study found that customizing ventilation settings for individual patients led to lower estimated hospital mortality rates compared to actual rates. This highlights the potential effectiveness of using reinforcement learning methodology to develop AI models that analyze complex clinical data for optimizing treatment parameters. Additionally, our findings suggest the integration of this model into a clinical decision support system for refining ventilation settings, supporting the need for prospective validation trials.

## Introduction

Mechanical ventilation is the foundation of critical care medicine and is one of the most common interventions for patients admitted to intensive care units (ICUs). Studies showed that approximately one-third of ICU patients require mechanical ventilation in the United States [1]. In recent years, due to the COVID-19 pandemic and aging populations in many countries, mechanical ventilation in ICU use has been constantly increasing.

Despite decades of research, choosing the optimal ventilator strategy for a patient remains imprecise. Appropriate ventilator settings are important but complicated by significant interpatient variability. Current clinical guidelines provide one-size-fits-all recommendations but do not personalize the treatment for different ICU patients. In particular, existing clinical guidelines do not address personalized optimal settings for mechanical ventilation, including positive end-expiratory pressure (PEEP) level, fraction of inspired oxygen (FiO_2_), and ideal body weight–adjusted tidal volume [2]. With the understanding that mechanical ventilation itself can cause and potentiate lung injury, it is important to choose appropriate ventilatory strategies to mitigate ventilator-induced lung injury [3]. Nonetheless, even guideline recommendations may not be adhered to, as a wide discrepancy in practice exists and evidence-based interventions are underused for the task [4].

The drive to discover an effective solution capable of managing the intricate ICU environment and providing personalized treatment to each patient is a compelling motivator. One particularly promising approach is the use of reinforcement learning (RL) for formulating treatment recommendations, supported by the following reasons. First, RL is a decision-making tool that can learn complex sequential decisions, making it a natural fit for critical care applications. Second, RL can take individual patients’ health conditions and disease histories into account, hence providing more personalized treatment decisions that have the potential to surpass existing clinical practices. However, the RL method for mechanical ventilation guidance needs further evaluation before committing resources for prospective clinical studies. We therefore aimed to test the concept that RL can optimize ventilator settings for patients on invasive mechanical ventilation, by applying RL on existing large ICU databases.

## Methods

### Overview of the Methods

**Figure 1:**
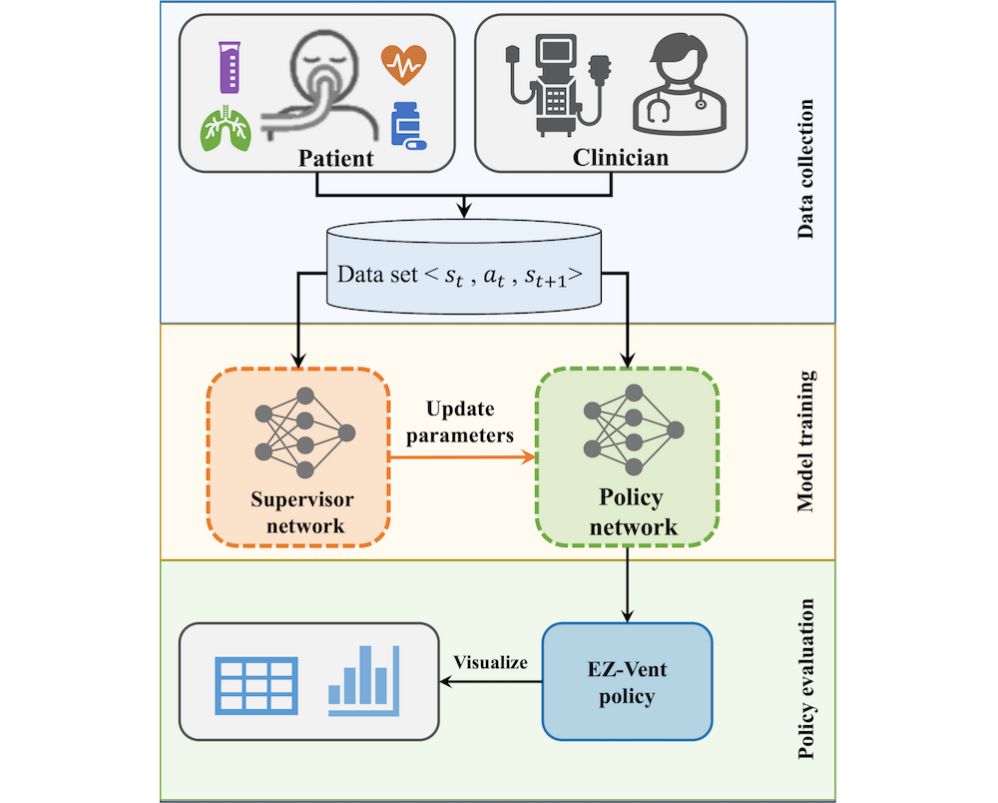
Proposed EZ-Vent framework. We collected data on ventilated patients from EHR and trained a policy to recommend optimal ventilator settings. EHR: electronic health record.

For our study, we named the RL-based artificial intelligence (AI) solution “EZ-Vent.” The framework of the proposed solution is shown in Figure 1. We first collected mechanically ventilated patients’ health data and intensivists’ treatment actions from 2 large electronic health record (EHR) datasets in the United States. We then trained a type of RL-based model, named the Batch Constrained Deep Q-learning (BCQ), to learn from physicians’ treatment actions and to develop an optimal strategy for setting mechanical ventilation. This type of learning is commonly referred to as batch learning in RL. However, many traditional RL algorithms have been unsuccessful in the batch setting, while the models they produced often suffered from overestimation and exhibited poor performance when presented with data not included in the provided batch. In contrast to traditional RL algorithms, the BCQ algorithm imposes constraints to ensure that the learned policy remains reasonably close to physicians’ policy. For this reason, we chose to implement BCQ in our solution due to its capacity to develop a safe policy from observational data. Given the crucial significance of safe policy learning in health care applications, the proposed AI solution may then be integrated as a component of a clinical decision support system, assisting intensivists in making optimal decisions for critically ill patients who require mechanical ventilation.

Our proposed AI solution recommends optimal ventilator settings for PEEP, FiO_2_, and tidal volume levels by considering the individual patients’ conditions including their demographic features, physiological status, and multiple comorbidities. Compared to the existing guidelines, the proposed solution can adjust treatment recommendations based on changes in a patient’s condition. Moreover, we developed a set of flags designed to detect sudden changes in patients’ health and leveraged the timing of these flags to partition patients’ trajectories into discrete time-varying intervals. We anticipated that these timings correspond to critical decision points for physicians to intervene in practice. If our model were to be implemented at the bedside in real time, it has the potential to assist intensivists in making more informed and optimized decisions. Studies have reported ICU mortality rates as high as 86% to 97% for invasive mechanical ventilation [5-7], and improved ventilator settings would greatly benefit critically ill patients. As such, our proposed AI solution holds promise for improving patient outcomes in ICUs for those requiring mechanical ventilation.

Although general improvements in ICU outcomes and changes in ventilation practices over time would positively affect the model in the training process, the proposed BCQ model would automatically learn from the good practices and avoid bad practices to achieve the best long-term return, which is the survival of the patients.

### Study Population and Datasets

The observational data for mechanically ventilated patients were extracted from 2 large EHR databases in the United States: the Medical Information Mart for Intensive Care IV (MIMIC-IV) database [8] and the e-Intensive Care Unit Collaborative Research (eICU) database [9]. We included patients who were aged 16 years and older, and whose ventilation duration was at least 24 hours. Only the first ICU admission for each patient was considered, and we studied the first 48 hours of ventilation data.

We excluded patients who did not have data for mortality, height, or sex. In addition, we excluded patients whose mechanical ventilation duration exceeded 2 weeks, because patients who required long-term mechanical ventilation may not be representative of the general population of patients who require mechanical ventilation. Lastly, we excluded patients who have missing ventilation settings of PEEP, FiO_2_, and tidal volume recorded for the entire ventilation duration.

**Figure 2:**
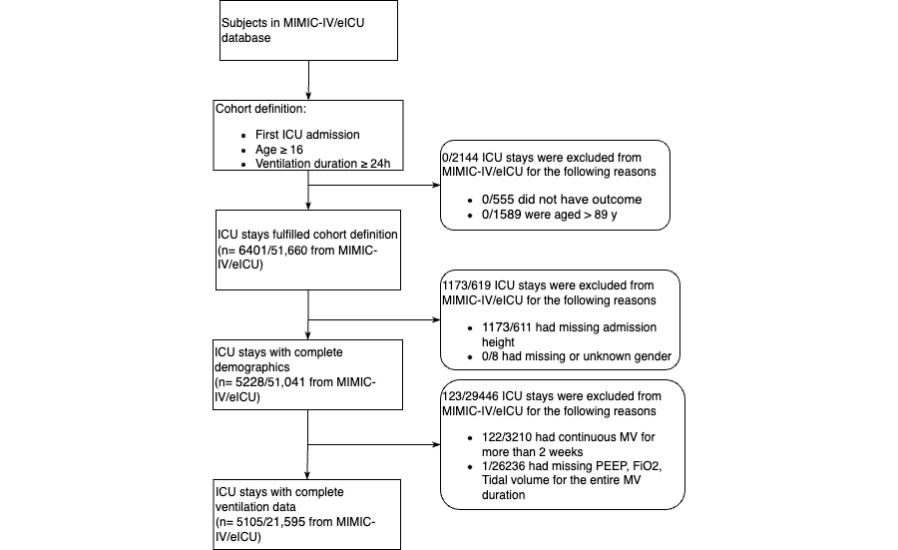
Overview of exclusion criteria and the number of patients left after each round of selection. eICU: e-Intensive Care Unit Collaborative Research; FiO2: fraction of inspired oxygen; ICU: intensive care unit; MIMIC-IV: Medical Information Mart for Intensive Care; MV: mechanical ventilation; PEEP: positive end-expiratory pressure.

After those exclusions, 5105 patients in the MIMIC-IV dataset and 21,595 patients in the eICU dataset remained. The flowchart for cohort selection is shown in Figure 2. We conducted a 5-fold cross-validation on the eICU dataset. The full MIMIC-IV dataset was held out as the testing set.

### Outcome Variables

The primary outcome was hospital mortality. Mortality outcomes are the most important outcomes for patients in the ICUs, given the high mortality for patients in ICUs in general. Mortality is a definitive measure of success in interventions, given the ultimate goal of ICU care is to save lives.

Secondary outcomes were the proportion of optimal oxygen saturation (SpO_2_) and the proportion of optimal mean arterial blood pressure (MBP). The optimal ranges of SpO_2_ and MBP were defined as follows: 94%<SpO_2_<98% [10] and 70 mm Hg≤MBP≤80mm Hg [11].

### RL: A Primer

RL is a goal-oriented AI method where a computer agent, acting as a decision maker, analyzes available data within its defined environment, derives a policy for taking actions, and optimizes long-term rewards. The agent is the computational model we want to develop. In general, an agent obtains evaluative feedback (reward) about the performance of its action at each consecutive time step, allowing it to improve the performance of subsequent actions by trial and error. Mathematically, the sequential decision-making process is called the Markov Decision Process (MDP). We define the 5-tuple MDP as (*S*, *A*, *P*, *R*, γ):

- State: A state *s_t_* ∈ *S* is the state at time *t* in state space *S*. In this study, it represents the health status of a patient at each timestamp. We constructed the patient’s state by using 40 relevant physiological features containing demographics, laboratory values, and vital signs (see Table S1 in Multimedia Appendix 1 for the full list).
- Action: An action *a_t_* ∈ *A* is the treatment option that the agent takes at each time step *t*, which influences the next state *s_t+1_*. In our study, the action space was constructed as 18 possible discrete actions from combinations of low, medium, and high levels of the 3 ventilator settings: PEEP, FiO_2_, and ideal body weight–adjusted tidal volume (Figure S1 in Multimedia Appendix 1.
- Transition probability: *P(s_t_|a_t_)* → *s_t+1_* is the probability of transiting from the state *s_t_* to the next state *s_t+1_* given an action *a_t_*.
- Reward: *R* is the observed feedback given a state-action (*s_t_, a_t_*) pair. The reward of our model reflected the objective of an RL agent, which was to improve survival and achieve oxygen saturation and mean arterial pressure within their respective optimal ranges. Hospital mortality was used as the terminal reward, whereas SpO_2_ and MBP were applied as intermittent rewards.
- γ ∈ {0,1} is the discount factor.

We assume the process of ventilator adjustment has Markov property. That is, the state space is completely observable, the state transition probability *P* is only related to the last state and the last action, and the immediate reward is only related to the state and the action taken in the corresponding step.

The solution of the MDP is an optimized set of rules, that is, the RL policy. The ability of RL to learn complex sequential decisions makes it suitable for critical care applications, and we hope to use its capability of learning to provide individualized treatment policies that could improve the survival of patients who are mechanically ventilated.

Specifically, we aim to train an RL policy π: *S* × *A* {0,1}, which specifies the probability of taking each action in each state through Q-learning, where *Q*^π^(*s*, *a*) is the value of taking action *a* in state *s* using policy π and is defined as the expected sum of future rewards, discounted by *γ* at each time step as follows:

*Q*^π^(*s*, *a*) = *E*{*r_t_*_+1_ + *γr_t_*_+2_ + *γ*^2^*r_t_*_+3_ + ... |π(*s*, *a*)}

### RL: Time-Varying Intervals and Flags

We applied time-varying intervals to discretized state and action pairs. For each patient’s treatment trajectory, we analyzed the data from the first hour of using mechanical ventilation until the 48th hour or until ventilator weaning, whichever came earlier. Then we cut the trajectories into 4-hour time steps, except for cases when flags were raised to further cut the trajectories. We designed the set of flags as follows: (1) the SpO_2_ dropped under 90%, (2) the partial pressure of oxygen (PaO_2_) dropped under 60 mm Hg, and (3) pH <7.25 or pH >7.45. When any flag was raised, the trajectories would be further cut into shorter time steps. We designed this set of flags to reflect real-world conditions, where such flags would prompt changes in ventilator settings. Next, we selectively merged time steps if they were too short, with a minimum time interval of no less than 1 hour. For multiple values in 1 time step, we computed a time-weighted average value. Patients that had at least 1 of the 3 actions empty for all the time steps were removed.

Data imputation is common in EHR data analysis as some data are manually entered by the doctors, which may cause them to be recorded less frequently. Similar ways such as k-nearest neighbor imputation and time-windowed sample-and-hold method have been applied to handle data imputation in previous works [12,13]. In the literature that reported the percentage of imputed data, the percentage of imputation was as high as 95% [13]. In our study, 66.28% (n=14,314/21,595) of patients in the eICU database had data requiring imputation. Among these patients, a median 87% (IQR 64%-95%) of data were imputed. Data in the MIMIC-IV database were more complete, with 33.8% (n=1724/5105) of patients requiring imputation. Among these patients, a median 72% (IQR 47%-89%) of data were imputed. The distribution of data that require imputation was reported in Figure S6 for eICU and S7 for MIMIC-IV in Multimedia Appendix 1. Missing values were imputed with the nearest value before the time step, and if this was not available, missing values were imputed with the value from the next time step. Binary state variables were represented using 0 or 1. Continuous state variables were normalized or log-normalized to (0, 1) as appropriate.

### RL: Model Development

One major drawback of using historical EHR data to train an RL model is extrapolation error. The term extrapolation is a statistical technique for estimating values that extend beyond a particular collection of data or observations. Extrapolation error is caused by the mismatch between the data distribution in the offline dataset and future observations. To mitigate this problem, we applied the BCQ model in this study, which is an RL model that has the advantage over other RL algorithms in the batch setting by addressing the extrapolation error [14].

The BCQ model consists of 2 modules, a supervisor network and a policy network. The supervisor network is used to mimic the physicians’ policy from the observational data. The policy network fits the optimal policy under the constraint of the supervisor network, where only the actions likely to be taken in the observational data are considered and evaluated. The final optimized policy is then expected to lead to good future outcomes as well as to select a safe action.

In this study, the loss of the BCQ model is defined as the combination of 2 loss functions: *L* = *L_Q_* + *βL_P_*, where *L_Q_* is the value loss and is defined as:



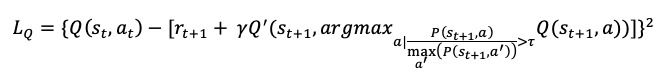



*L_P_* is the probability loss and is defined as *L_P_* = –log (*P*(*s_t_*, *a_t_*)). The final RL policy is defined as:



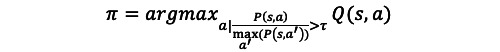



In the training stage, when BCQ receives the training sample, the supervisor network will first learn the mapping from state to action, that is, which action would be taken based on historical data. Then, the policy network will optimize its policy with the reward information and the output of the supervisor network. This training process is iterated several times until we derive the final AI policy.

For the supervisor network, we adopted a fully connected stream with 2 hidden layers of 256 units to infer the action value *Q*^π^(*s*, *a*) function and a fully connected stream with 2 hidden layers of 256 units to infer the state-action probability *P*(*s_t_*, *a_t_*). Each hidden layer contained the rectified linear unit activation. The policy network had the same structure as the supervisor network. The learning rate was 0.0003, the discount factor *γ* was 0.99, the batch size was 32, the tracking rate α was 0.01, the extrapolation threshold τ was 0.05, and the trade-off factor of 2 kinds of loss functions β was 1. We trained the RL model using the Adam optimizer.

We designed a clinically guided reward function that produced a reward (penalty) when the patients’ state improved (deteriorated) based on short-term and long-term health outcomes. The short-term health indicators were the patients’ MBP as well as SpO_2_. At each intermittent (ie, nonterminal) time step of a patient’s trajectory, the patient would receive a positive short-term reward *b* if MBP fell within the range of 70-80 mm Hg ] or *c* if SpO_2_ fell within 94% to 98% based on the literature on maintaining optimal levels of vital signs and blood gases, and the patient would receive a penalty (negative reward) of –*b*/2 or –*c*/2 when MBP and SpO_2_ fell out of the range. We applied the range (94%, 98%) of SpO_2_ for the intermittent reward design due to the following reasons: conservative O_2_ therapy has been variably defined in various randomized controlled trials (RCTs). RCTs investigating the lower SpO_2_ threshold have found evidence of increased mortality from SpO_2_ <93% [15]. Other RCTs did not show any increased mortality in their conservative groups if these groups attained an SpO_2_ (or equivalent PaO_2_) within 94% to 98% (eg, a conservative group of ICU randomized trial comparing 2 approaches to oxygen therapy [16] had time-weighted PaO_2_ ~80 mm Hg and the lowest SpO_2_ group of Pragmatic Investigation of Optimal Oxygen Targets [17] had SpO_2_ around 94%). At terminal time steps, each patient would receive a final reward *a* (or penalty –*a*/2) if a patient survived (or became deceased) at discharge. The overall reward function was defined as follows:



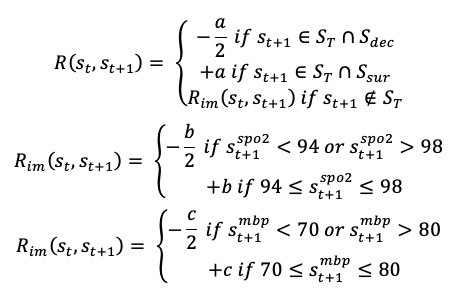



where *S_T_*, *S_sur_*, and *S_dec_* sets represented terminal, survived, and deceased patient states, and *a, b,* and *c* were parameters that were tuned during training. The reward information at each time step would help BCQ learn those action patterns from physicians that lead to good short-term and long-term outcomes.

To account for potential cointerventions that would affect the MBP and survival, we included the maximum dose of vasopressor (Table S1 in Multimedia Appendix 1) over every 4-hour time window of mechanical ventilation within patient states for our RL model. In addition, as differences in illness severity could also modify mortality risk, we included the patients’ Elixhauser score, sequential organ failure assessment (SOFA) score, number of systemic inflammatory response syndrome criteria, vital signs, and laboratory test results (Table S1 in Multimedia Appendix 1) in the patient state to reflect differences in patients’ illness severity over time. The treatment action from the model was conditioned on all the state variables so that state differences were handled in the model.

### RL: Benchmark Policies

We evaluated our RL-based policy by comparison with 3 benchmark policies:

- Random policy: All 18 discrete actions have equal probabilities to be chosen.
- One-size-fits-all policy: The action with the highest probability in the cohort is always chosen.
- Physicians’ policy: The actual observed policy in the validation and testing sets.

### RL: Evaluation Metrics

We used extensive quantitative and qualitative analyses to evaluate the performance of the learned AI policy and benchmarks. First, to understand the relationship between the expected return of the learned policies and the clinical outcomes, we mapped the expected return to the estimated outcome occurrence. We sorted the expected returns of the physicians’ policy into discrete bins and obtained the average empirical mortality rate from the patients in each bin. The empirical mortality estimate was used to derive a relationship between the range of computed returns of the AI policy against the observed mortality. This estimation process was performed for secondary outcomes too.

Treatment recommendation is an off-policy learning problem, which aims to learn an optimal policy using trajectories from an observed behavior policy (physicians’ policy). Evaluating the learned policy with off-policy estimation (OPE) methods is crucial for health care applications to avoid the high risk of failure or negative impact. OPE methods use examples from the behavior policy to evaluate the performance of the learned policy. Precise evaluation with OPE remains a challenging problem. Previous studies have used the V-curve method [18,19], observed mortality [19,20], importance sampling evaluation [21], and eligibility traces [22]. In this work, we adopted multiple evaluation metrics. We followed the V-curve method to qualitatively evaluate changes in mortality with action differences. We also quantitatively estimated the mortality rate and performed importance sampling with one type of importance sampling estimator, namely Consistent Weighted Per-Decision Importance Sampling (CWPDIS) [23], which is defined as:



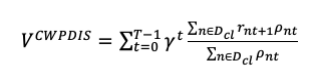



where *D_beh_* is a retrospective trajectory set generated by physician policy π*_cl_*, and *n* = (*S_n_*_0_, *a_n_*_0_, *r_n_*_1_, *s_n_*_1_, *a_n_*_1_, *r_n_*_2_, ..., *s_nT_*_–1_, *a_nT_*_–1_, *r_nT_*) is a specific trajectory with state, action, and reward in each time step. Note that CWPDIS is based on important sampling, which is a general technique for accomplishing OPE. Compared with other OPE methods, CWPDIS could make unbiased evaluations with higher sampling efficiency. In addition, we used a random forest classification model to rank the importance of various predictors for the actions under the physicians’ policy (Figures S3-S5 in Multimedia Appendix 1). This allowed us to understand physicians’ behavior regarding the choice of ventilator settings.

### Ethical Considerations

The collection of patient information and creation of the research resource in the MIMIC-IV database was reviewed by the Institutional Review Board at the Beth Israel Deaconess Medical Center (number 2001-P-001699/14), which granted a waiver of informed consent and approved the data-sharing initiative. For the eICU database, it has been approved by the Institutional Review Board of the Massachusetts Institute of Technology. After completing the National Institutes of Health’s online training course and the Protection of Human Research Participants Examination, we had the access to extract data from both the MIMIC-IV and the eICU databases. The study data are anonymous and deidentified.

## Results

### Patient Characteristics

Patient characteristics of the selected cohorts from the MIMIC-IV and eICU datasets are provided in Table 1. There were no statistically significant differences in age, sex, or body weight. However, we observed that patients in the MIMIC-IV dataset had greater illness severity compared with those in the eICU dataset. Patients in the MIMIC-IV dataset had higher Elixhauser scores (5.0 [0.0, 12.0] vs 3.0 [0.0, 7.0]), higher reintubation rate (30.7% vs 16.7%), longer hospital stay (291.0 hours [171.0, 477.0] vs 191.4 hours [120.1, 307.5]), and higher hospital mortality rate (31.1% vs 18.2%) compared to patients in the eICU dataset.

**Table 1:** Patient characteristics.

Variables	MIMIC-IV (N=5105)	eICU (N=21,595)
Female (%)	2154 (42.2)	9244 (42.8)
Age (years), median (IQR)^a^	65.0 (53.0, 76.0)	64.0 (53.0, 74.0)
Body weight (kg), median (IQR)^a^	80.9 (67.6, 97.2)	81.9 (68.0, 99.7)
Reintubation (%)	1565 (30.7)	3661 (16.7)
Elixhauser score, median (IQR)^a^	5.0 (0.0, 12.0)	3.0 (0.0, 7.0)
First SOFA^b^ score, median (IQR)^a^	2.0 (0.0, 4.0)	2.0 (0.0, 4.0)
Hospital LOS^c^ (h), median (IQR)^a^	291.0 (171.0, 477.0)	191.4 (120.1, 307.5)
Hospital mortality (%)	1590 (31.1)	3934 (18.2)
PEEP^d^, (cmH2O), median (IQR)^a^	5.3 (5.0, 10.0)	5.0 (5.0, 5.0)
FiO_2_ (%), median (IQR)^a^	50.0 (40.0, 60.0)	49.2 (40.0, 61.1)
Tidal volume^e^ (ml/kg IBW^f^), median (IQR)^a^	7.2 (6.4, 8.3)	7.8 (6.9, 8.8)

^a^25th percentile, 75th percentile.

^b^SOFA: sequential organ failure assessment.

^c^LOS: length of stay.

^d^PEEP: positive end-expiratory pressure.

^e^Tidal volume: ideal weight-adjusted tidal volume.

^f^IBW: ideal body weight.

### Performance of the RL Method

**Figure 3:**
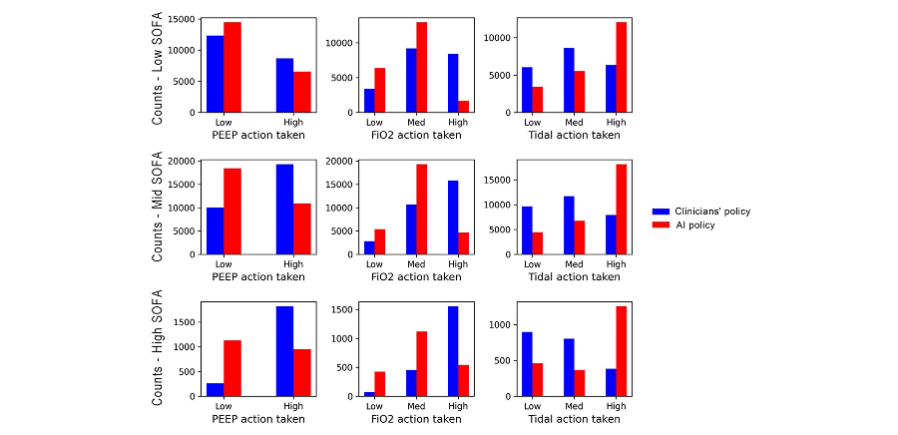
Comparative action distributions for physicians (blue) and learned policy (red) in the MIMIC-IV test set. Each panel represents actions taken for different SOFA score levels: low (SOFA<5), medium (5≤SOFA<15), and high (SOFA>15). Actions taken by physicians were different from those suggested by AI. The learned policy recommended more low-level actions for PEEP and FiO2 and high-level actions for tidal volume. AI: artificial intelligence; FiO2: fraction of inspired oxygen; Med: medium; Mid: middle; MIMIC-IV: Medical Information Mart for Intensive Care; PEEP: positive end-expiratory pressure; SOFA: sequential organ failure assessment.

**Figure 4:**
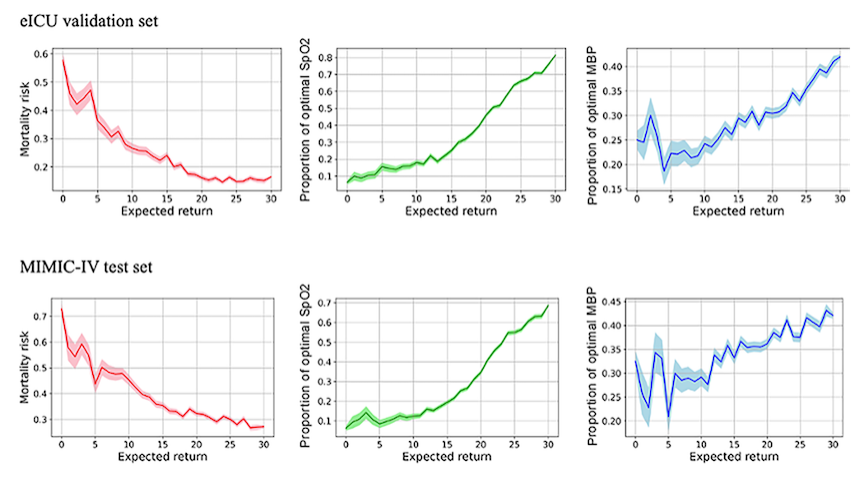
Changes in observed mortality (red), proportion of optimal SpO2 (green), and proportion of optimal MBP (blue) versus the expected return curves for learned policies in the eICU validation set and MIMIC-IV test set. The proportion of mortality (red) against the returns showed inverse relationships, whereas the proportion of optimal SpO2 (green) and the proportion of optimal MBP (blue) against returns showed overall positive relationships in both data sets. Overall, the figures show that policies with higher returns were associated with lower mortality and higher proportions of optimal SpO2 and MBP. eICU: e-Intensive Care Unit Collaborative Research; MBP: mean arterial blood pressure; MIMIC-IV: Medical Information Mart for Intensive Care; SpO2: optimal oxygen saturation.

We plotted the action frequency distributions of the physicians’ policy and the learned AI policy. We compared the learned policy against the physicians’ policy for low (<5), medium (5-15), and high (>15) SOFA score levels for patients in the eICU (Figure 3) and the MIMIC-IV databases (Figure S3 in Multimedia Appendix 1). For each SOFA group, we counted the number of actions for the 3 action categories: PEEP, FiO_2_, and tidal volume. Actions taken by physicians were different from those suggested by AI. The learned policy recommended more low-level actions for PEEP and FiO_2_ and high-level actions for tidal volume. Relationships between the range of computed returns of the learned policy against various outcomes for both the eICU validation set and the MIMIC-IV test set are shown in Figure 4. The figures show that policies with higher returns were associated with lower mortality and higher proportions of optimal SpO_2_ and MBP.

**Figure 5:**
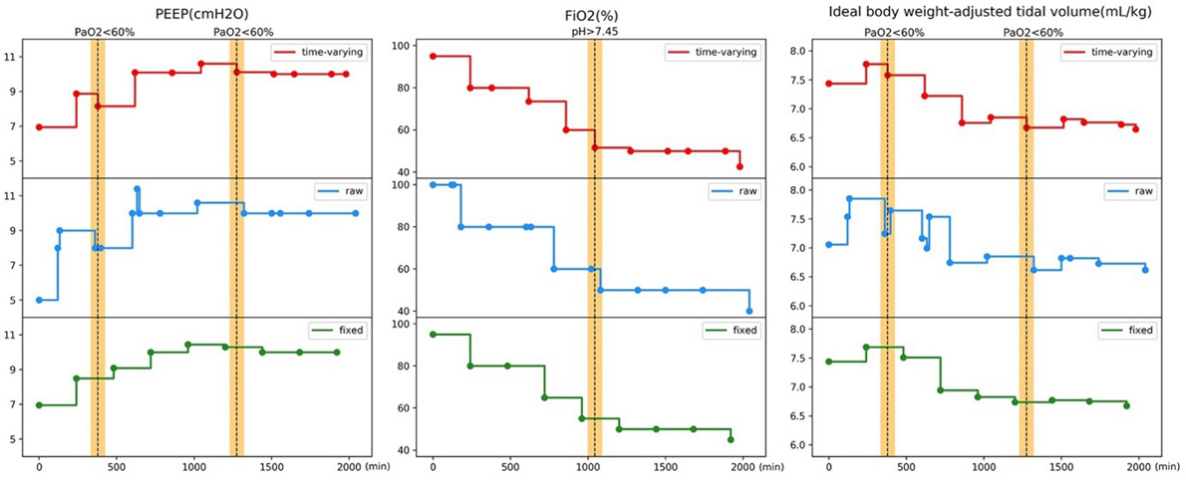
Visualization of representative patient cases in raw, fixed, time-varying intervals for mechanical ventilator action setting. Visualization of representative case study for mechanical ventilator settings of PEEP (left), FiO2 (middle), and ideal body weighted-adjusted tidal volume (right) using time-varying interval (red), raw data (blue), and fixed 4-hour time interval (green). Flags to cut intervals in the time-varying setting are shown as vertical dotted lines with yellow shadows. The flags could catch the changes in the ventilator settings. FiO2: fraction of inspired oxygen; PaO2: partial pressure of oxygen; PEEP: positive end-expiratory pressure.

**Figure 6:**
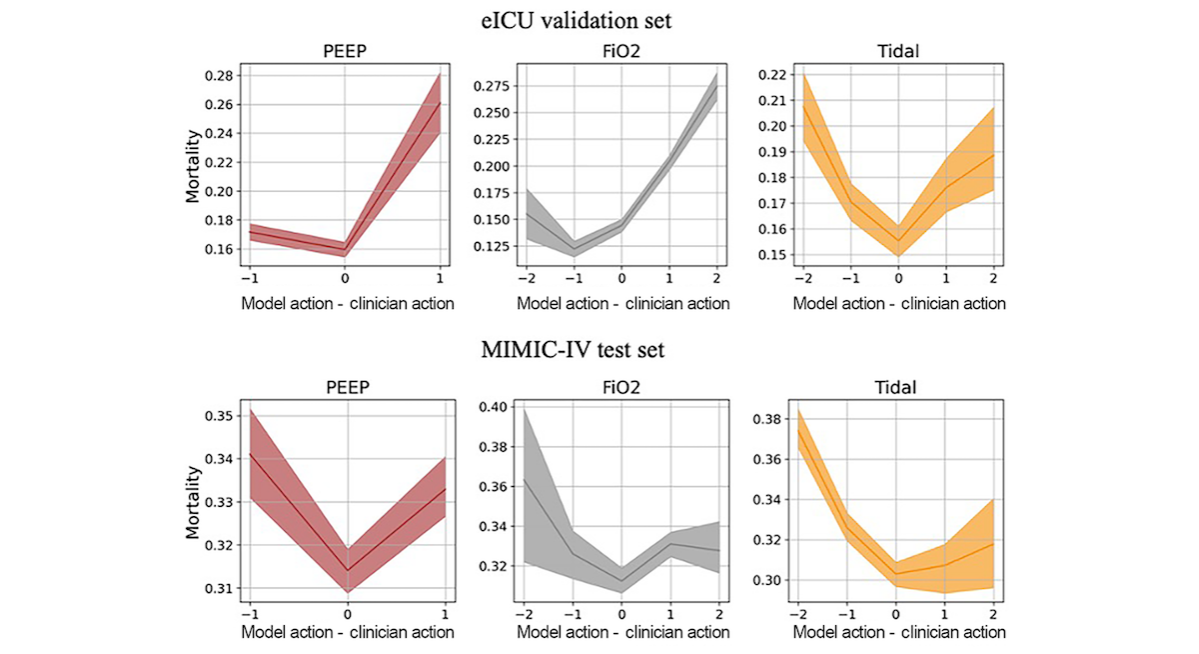
Changes in observed mortality (y-axis) versus the difference between the mechanical ventilation settings recommended by the optimal policy and the settings administered by physicians (x-axis) on the eICU validation set and MIMIC-IV test set. The x-axis indicates the differences in the quantile number. The plots show a v-shape which indicates that mortality is the minimum when we follow the actions suggested by the policy. eICU: e-Intensive Care Unit Collaborative Research; FiO2: fraction of inspired oxygen; MIMIC-IV: Medical Information Mart for Intensive Care; PEEP: positive end-expiratory pressure.

The OPE performance of the learned policy is shown in Table 2. We estimated the hospital mortality rate (eICU 12.1%, SD 3.1%; MIMIC-IV 29.1%, SD 0.9%), the proportion of optimal SpO_2_ (eICU 58.7%, SD 4.1%; MIMIC-IV 49%, SD 1%), and the proportion of optimal MBP (eICU 31.1%, SD 4.5%; MIMIC-IV 41.2%, SD 1%) for the learned policy. We also report outcomes for the physicians’ policy, including the observed mortality rate (eICU 14.3%; MIMIC-IV 30.6%), the proportion of optimal SpO_2_ (eICU 47.8%; MIMIC-IV 40.5%), and the proportion of optimal MBP (eICU 28.2%; MIMIC-IV 37.1%) in the 2 datasets, respectively. We also performed *t* tests (2-tailed) for proportions of optimal SpO_2_ and MBP, and Fisher exact tests for hospital mortality rate, and calculated the *P* values compared with the physicians’ policy. The results from all 3 policies achieved *P* values of <.001, which indicates that the differences were very unlikely to arise from randomness. Overall, the AI policy achieved a longer duration within optimal SpO_2_ and MBP ranges with lower mortality compared to the physicians’ policy. To examine the effectiveness of using time-varying intervals in the action setting, we visualize a representative patient case in Figure 5. The relationship between mortality and discrepancy between AI and physicians’ ventilator settings is illustrated in Figure 6.

We report the feature importance with regards to choosing ventilator settings under the physicians’ policy in Figures S3-S5 in Multimedia Appendix 1. The top 10 important features included the following: PaO_2_/FiO_2_ ratio, PaCO_2_, PaO_2_, creatinine level, lactate, prothrombin time, base excess, age, admission weight, and Richmond Agitation Sedation Scale score.

**Table 2:** Outcomes using the validation set (eICU^a^) and test set (MIMIC-IV^b^).^c^

Dataset	Policy	Proportion of time within SpO_2_^d^ range (%), mean (SD)	Proportion of time within MBP target^e^ range (%), mean (SD)	Observed mortality (%), mean (SD)	*P* value	95% CI
**eICU**						
	Physician^f^	47.8 (5.0)	28.2 (4.5)	14.3 (3.6)	—^g^	—
	Random^h^	49.9 (4.8*)	30.7 (4.5*)	15.2 (3.5*)	<.001	15.17-15.23
	One-size-fit-all^i^	53.2 (4.9*)	29.4 (4.5*)	17.5 (3.8*)	<.001	17.47-17.53
	AI^j^	58.7 (4.7*)	31.1 (4.5*)	12.1 (3.1*)	<.001	12.07-12.13
**MIMIC-IV**						
	Physician	40.5 (1.0)	37.1 (1.0)	30.6 (0.9)	—	—
	Random	34.6 (1.0*)	36.2 (1.0*)	32.3 (0.9*)	<.001	32.29-32.31
	One-size-fit-all	40.9 (1.0*)	38.5 (1.0*)	32.0 (0.9*)	<.001	31.99-32.01
	AI	49.0 (1.0*)	41.2 (1.0*)	29.1 (0.9*)	<.001	29.09-29.11

^a^eICU: e-Intensive Care Unit Collaborative Research.

^b^MIMIC-IV: Medical Information Mart for Intensive Care IV.

^c^*P value <.001 when compared to physicians’ policy.

^d^SpO_2_ target range: 94%<SpO_2_<98%.

^e^MBP target range: 70 mm Hg ≤ MBP ≤ 80 mm Hg.

^f^Physician: the actual observed policy in the validation and testing set.

^g^Not applicable.

^h^Random: all the 18 discrete actions have equal probabilities to be chosen.

^i^One-size-fit-all: the action with the highest probability in the cohort is always chosen.

^j^AI: artificial intelligence policy from Batch Constrained Deep Q-learning model.

## Discussion

### Overview

In this study, we used an RL-based AI model (BCQ) to learn the optimal ventilation policy customized for patients who are critically ill and require mechanical ventilation. We validated the policy using 2 large public datasets from the United States: the eICU and MIMIC-IV datasets. In both datasets, the learned policy had superior performance compared to the observed physicians’ policy, based on several quantitative and qualitative evaluation metrics.

### Principle Findings

In the MIMIC-IV dataset patients exhibited a higher severity of illness relative to those in the eICU dataset. However, this presented an opportunity to evaluate the extrapolation capacity of the BCQ model. The BCQ model-derived RL policy consistently demonstrated superior performance to physicians’ policy in both datasets. Consequently, we surmised that the BCQ model’s extrapolation ability was acceptable.

We formulated the clinical problem of choosing optimal ventilator settings in the ICU as an RL problem. We then used relevant physiological variables to represent patients’ health status as states and cut the ventilator treatment trajectories into time-varying steps to reflect the changes in patients’ conditions. We designed a set of flags to capture the sudden changes in patients’ health and used the flag timings to further cut the trajectory because such timings were the likely decision points for physicians to make necessary interventions. From the visualization of time-varying intervals in Figure 5, we observed that when the flags were raised (vertical dotted line), time-varying interval setting (red lines) can better reflect the changes in raw data (blue lines) of ventilator settings promptly compared to fixed 4-hour time intervals (green lines).

The AI policy used a “penalty” and “reward” function to regulate SpO_2_ and MBP within their optimal ranges. Notably, the policy tried to avoid hyperoxemia due to evidence of its harmful effects, as demonstrated by randomized trials in adults and younger patients [24,25]. While evidence of harm in hypertension was not as strong as in hyperoxemia, physicians could avoid overdosing on vasopressors to minimize the risk of arrhythmia [26]. Nonetheless, caution should be exercised in the use of the model, and it should not be relied upon as a standalone tool. On the contrary, it was designed as a decision support tool that provides more personalized guidance and might lead to better treatment plans. Physicians should evaluate the recommendations from the model carefully and balance between AI predictions and established treatment protocols.

From the action frequency distribution plot (Figure 3) for patients in MIMIC-IV, we found that the actions from physicians (red) and the actions recommended by AI policy (blue) have some discrepancies in all ventilator settings. This result is desirable because the supervisor network in the BCQ model does not aim to duplicate physicians’ choices. On the contrary, the supervisor network was used to learn good action patterns from physicians and limit the choice of actions with constraints. In addition, we found the learned policy recommended low-level PEEP and high-level ideal body weight-adjusted tidal volume more frequently compared to physicians’ current practices for all the SOFA groups. This finding suggests that the high PEEP-low tidal volume strategy for acute respiratory distress syndrome [2,27] may not be optimal for all mechanically ventilated patients (eg, patients with focal acute respiratory distress syndrome [28]) and should not be applied as a one-size-fits-all approach. For the management of FiO_2_, the learned policy suggested more frequent use of low and medium levels and avoided high levels of FiO_2_ for all SOFA groups. This policy suggestion is in line with the known harm from excessive oxygenation, which has been found across different types of critical illness [10,29,30].

We computed the learned policy’s expected return, and we plotted it against mortality risk in Figure 4. We observed inverse relationships between expected return and mortality (red) in both validation and testing datasets. This indicates that the optimal policy (high return) results in lower mortality for patients. For the secondary outcomes related to maintaining SpO_2_ and MBP within their respective optimal ranges, the expected return showed positive relationships (green for SpO_2_ and blue for MBP). This indicates that the optimal policy (high return) leads to higher proportions of SpO_2_ and MBP within their respective optimal ranges.

Figure 6 highlights the mortality differences associated with discrepancies between AI-driven and clinician-determined ventilator settings. An effective policy has the lowest mortality when the recommended and administered ventilator settings coincide (the x-axis value is zero), indicating that when the practice strictly followed the AI policy, it had the lowest mortality. At the same time, for an effective policy, the observed mortality should increase as the administered ventilator settings deviate from the recommended settings of the AI policy. Accordingly, an effective policy should have a V-shaped curve with a minimum of 0, which we observed for the AI policy under all the 3 action groups (PEEP, FiO_2_, and tidal volume).

From the quantitative evaluation using CWPDIS, we found the learned policy had the lowest observed mortality compared with all 3 benchmark policies. At the same time, the learned policy achieved the highest proportion of optimal SpO_2_ and MBP in both datasets. Intuitively and as expected, the random policy had the worst outcome among all the policies.

### Comparison to Prior Work

Many recent works have provided RL methods to address treatment recommendation problems [18-21,31-33]. Deep Q Network is one of the most popular RL models used in the literature, which is a powerful model that can handle high-dimensional state spaces, and noisy and incomplete input data. However, the Deep Q Network would not perform well when certain states are rarely observed in the historical data, and it tends to make random treatment assignments in such scenarios. This is referred to as extrapolation error, when a model is used to make predictions outside the range of the input data during training. On the contrary, BCQ is an effective tool to avoid extrapolation error, because it is designed to be more robust to the distribution of the data. This is achieved using a regularization term that encourages the policy to remain close to the initial policy during training when rare states are observed. Other works [20,33] focus on the novel algorithms increasing the model complexity and do not pay enough attention to the extrapolation error.

In addition, previous RL methods used for ICU care used fixed 4-hour intervals in the action setting [18,19]. In this work, we propose a time-varying intervals setting to capture fine-grained treatment assignments, which is more in line with real-world clinical practice.

### Limitations

Although our study harnessed 2 large databases for derivation and external validation of an RL model, several limitations remain. First, the ICU environment is highly complex, with many interacting variables that may not be fully captured in the EHR data, thus making it challenging for the RL model to deliver accurate and effective policy. We tried to exploit the data using advanced modeling techniques to capture high-dimensional state spaces’ characteristics. Second, our study is retrospective, and the results require prospective validation to ensure safety before deploying it in the ICUs. Third, our study trained on a patient cohort in the United States, which is a high-income country with advanced medical care. Whether the RL model would perform similarly in a lower-resourced country is unknown and the model performance may not be generalizable to such resource-limited settings. Future validation should therefore be done in countries belonging to various World Bank income groups.

### Future Directions

Despite the above limitations, our study highlights the potential of AI (specifically RL) to personalize medical care by accounting for the myriad variations in patients’ clinical features and tailoring treatment recommendations according to those variations. By using the RL model, different actions could be used to quantify and compare the quality of the current treatment options for a given patient, concerning mortality rate. The proposed solution allows physicians to collaborate with the AI agent while retaining physician control of the decision-making process. Our method may also be applied to complex clinical decision-making beyond mechanical ventilation, such as sepsis management [21] and drug dosing [33].

To ensure the reliability and generalizability of our findings, we conducted thorough validation, both internally and externally, using 2 separate datasets. Despite these efforts, we acknowledge the limitation of using retrospective data to model clinical benefits. To confirm our preliminary results, randomized trials comparing AI-guided management with usual care and protocolized care can be done. The method of AI deployment can also be tested under different conditions: as an advisory versus strict implementation [34].

### Conclusions

The clinical implications of using RL models to suggest mechanical ventilation settings in ICU settings are of considerable importance. In this study, the RL model was trained to learn from patient data and to adjust mechanical ventilation settings, thereby optimizing patient outcomes. As the model is capable of continuously adapting to the patient’s evolving needs, the AI policy has the potential to outperform current clinical interventions and optimize personalized care for patients who are critically ill. One possible development is the integration of the AI agent into a clinical decision support system to optimize ventilation settings. However, before this can be done, prospective validation of this method will be needed in various ICU settings.

## Appendix

Multimedia Appendix 1 Tables and figures for features and action details.

## Abbreviations

AI: artificial intelligence
BCQ: Batch Constrained Deep Q-learning
CWPDIS: Consistent Weighted Per-Decision Important Sampling
EHR: electronic health record
eICU: e-Intensive Care Unit Collaborative Research
FiO2: fraction of inspired oxygen
ICU: intensive care unit
MBP: mean arterial blood pressure
MDP: Markov Decision Process
MIMIC-IV: Medical Information Mart for Intensive Care
OPE: off-policy estimation
PaO2: partial pressure of oxygen
PEEP: positive end-expiratory pressure
RCT: randomized controlled trial
RL: reinforcement learning
SOFA: sequential organ failure assessment
SpO2: optimal oxygen saturation

## Data Availability

The data that support the findings of this study are openly available in the MIMIC-IV Database [8] and the eICU database [9]. Our code is available online [35].
