# Supplementary material for: Reinforcement Learning to Optimize Ventilator Settings for Patients on Invasive Mechanical Ventilation: Retrospective Study
Source: J Med Internet Res. 2024 Oct 16;26:e44494. doi: 10.2196/44494 (PMC11525081; doi:10.2196/44494)
Supplement: Multimedia Appendix 1 [file jmir_v26i1e44494_app1.docx]

Table S1. List of state variables.

| Category | Feature Name |
| --- | --- |
| Demographics (5) | age, gender, weight, readmission to ICU, Elixhauser score |
| Vital signs and scores (11) | GCS, SOFA, SIRS, HR, SBP, DBP, MBP, respiratory rate, SpO2, RASS, temperature |
| Lab values (22) | BUN, glucose, INR, PTT, PT, pH, platelets count, calcium, ionized calcium, hemoglobin, lactate, creatinine, base excess, magnesium, WBC, PaCO2, ETCO2, bicarbonate, potassium, albumin, sodium, chloride |
| Medications and fluid balance (2) | urine output over 4h, maximum dose of vasopressor over 4h |

GCS: Glasgow Coma Scale; SOFA: Sequential Organ Failure Assessment Score; SIRS: Systemic Inflammatory Response Syndrome; HR: heart rate per minute; SBP: systolic blood pressure; DBP: diastolic blood pressure; MBP: mean blood pressure; RASS: Richmond Agitation-Sedation Scale; BUN: blood urea nitrogen; INR: International Normalized Ratio; PTT: Partial Thromboplastin Time; PT: Prothrombin Time; WBC: White blood cell count.

Figure S1. Categorization of action levels for PEEP, FiO2, and tidal volume. FiO2: fraction of inspired oxygen; PEEP: positive end-expiratory pressure.

**
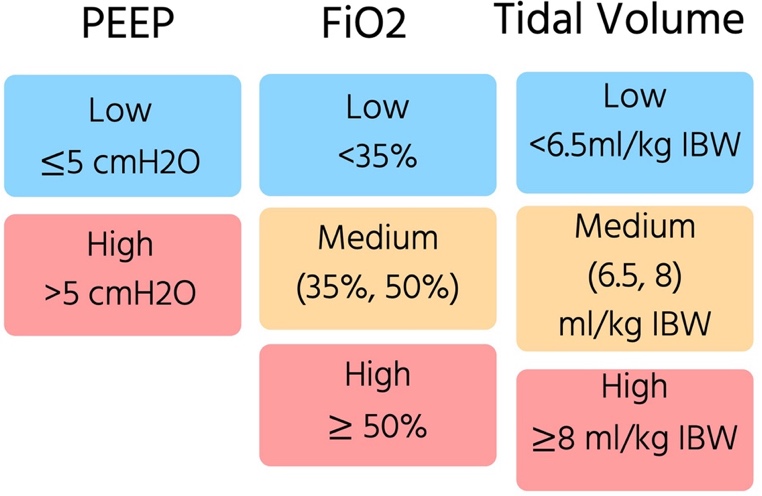
**

Figure S2. Action distributions for physicians (blue) and learnt policy (red) for patents with low/mid/high levels of SOFA score in eICU validation set. eICU: eICU Collaborative Research; SOFA: sequential organ failure assessment.


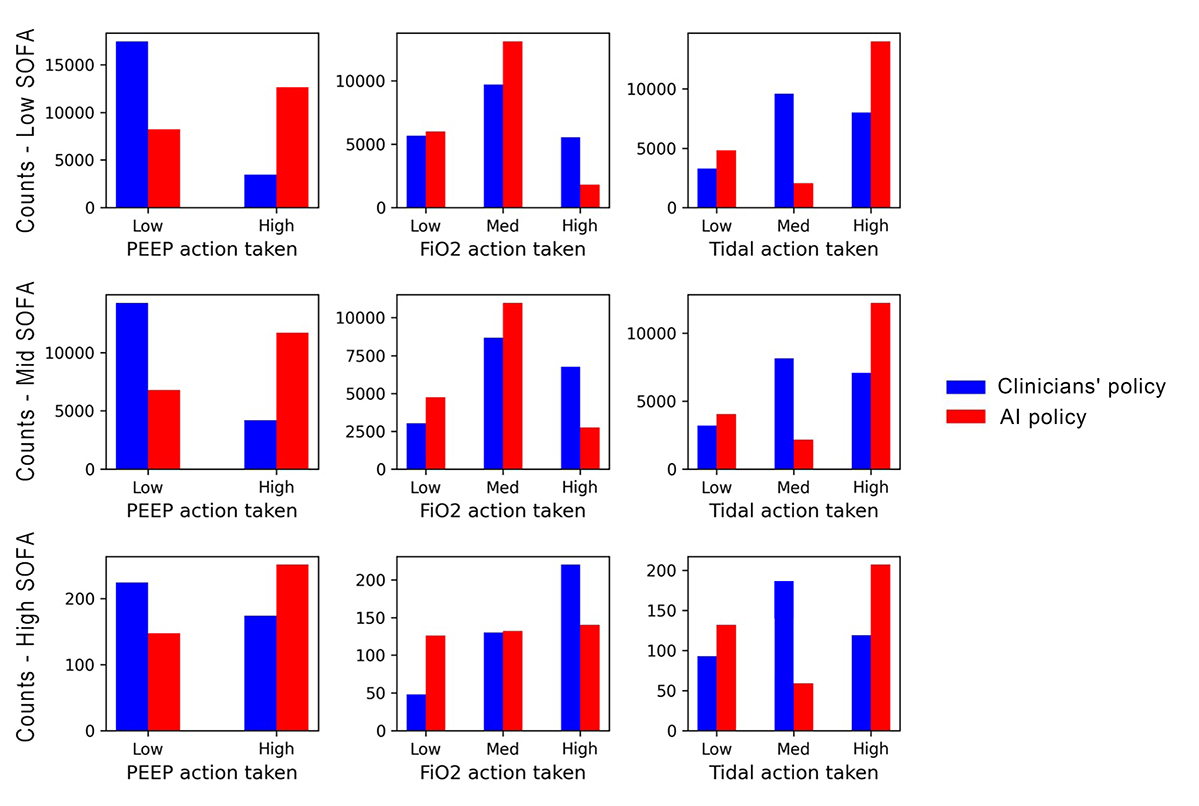


Note: Low SOFA: SOFA<5, Mid SOFA: 5$\leq$SOFA<15, High SOFA: SOFA>15

Figure S3. Feature importance for PEEP selection during mechanical ventilation. PEEP: positive end-expiratory pressure.

**
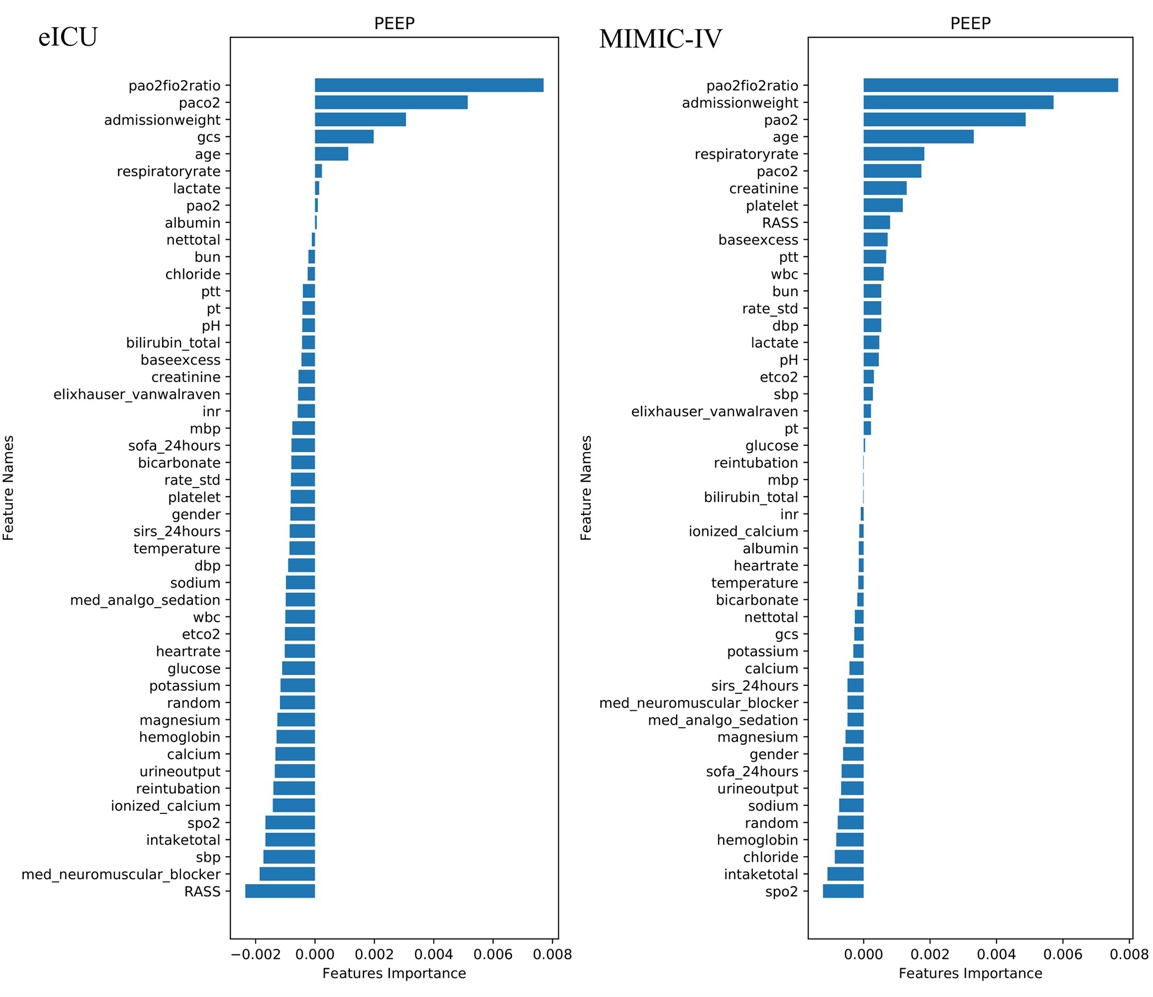
**

Figure S4. Feature importance for FiO2 selection during mechanical ventilation. FiO2: fraction of inspired oxygen.


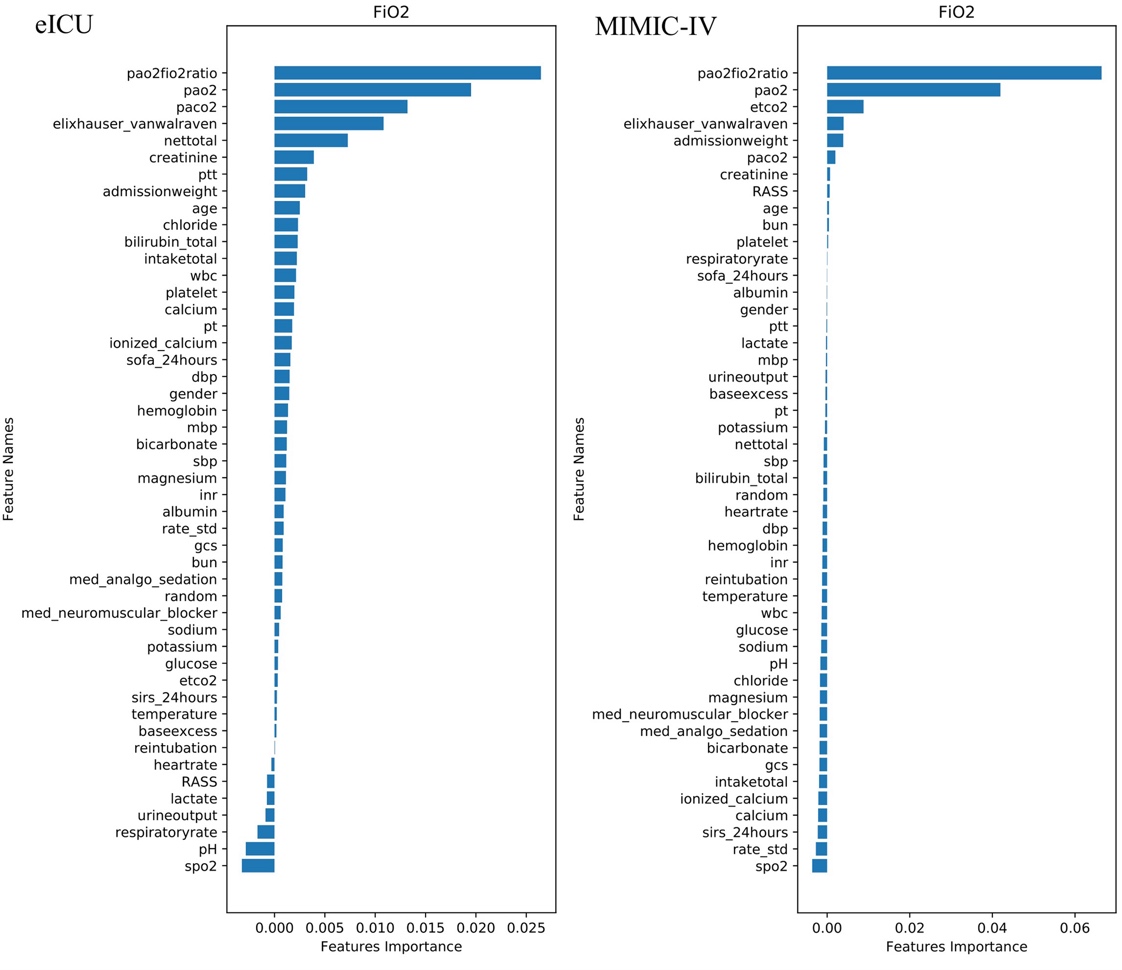


Figure S5. Feature importance for tidal volume selection during mechanical ventilation.

**
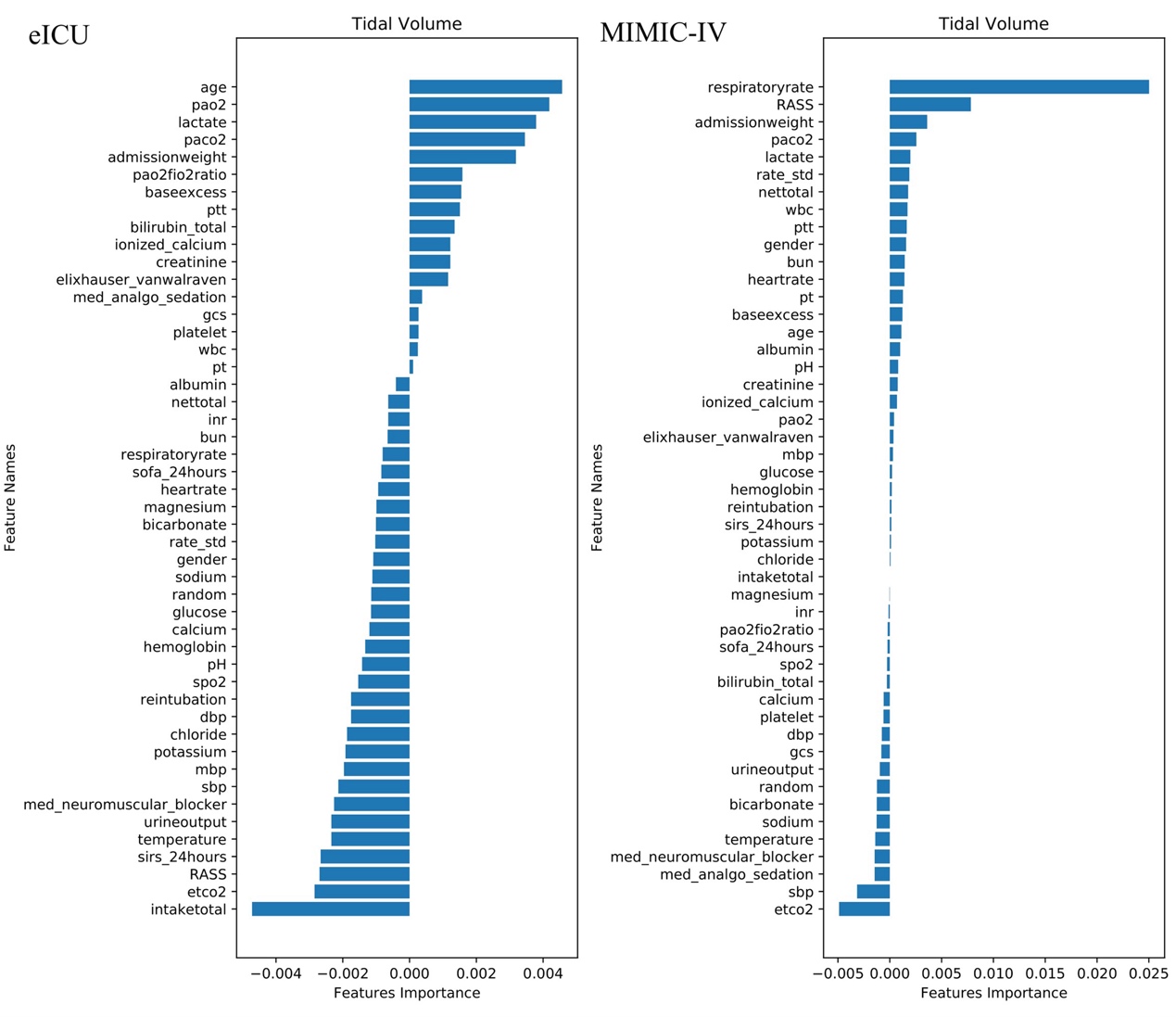
**

**Figure S6.** Distribution of empty values for eICU data set. eICU: eICU Collaborative Research.

**
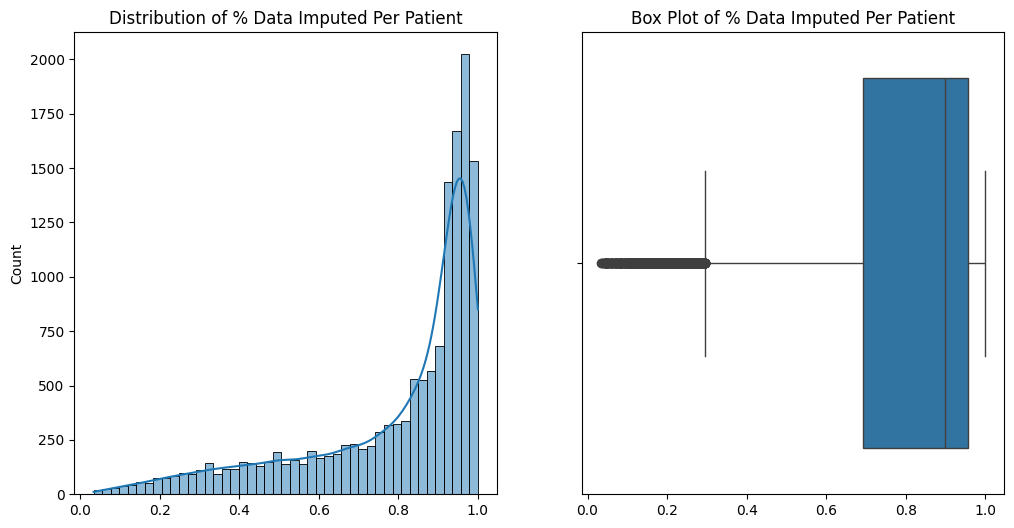
**

**Figure S7.** Distribution of empty values for MIMIC-IV data set. MIMIC-IV: Medical Information Mart for Intensive Care.

**
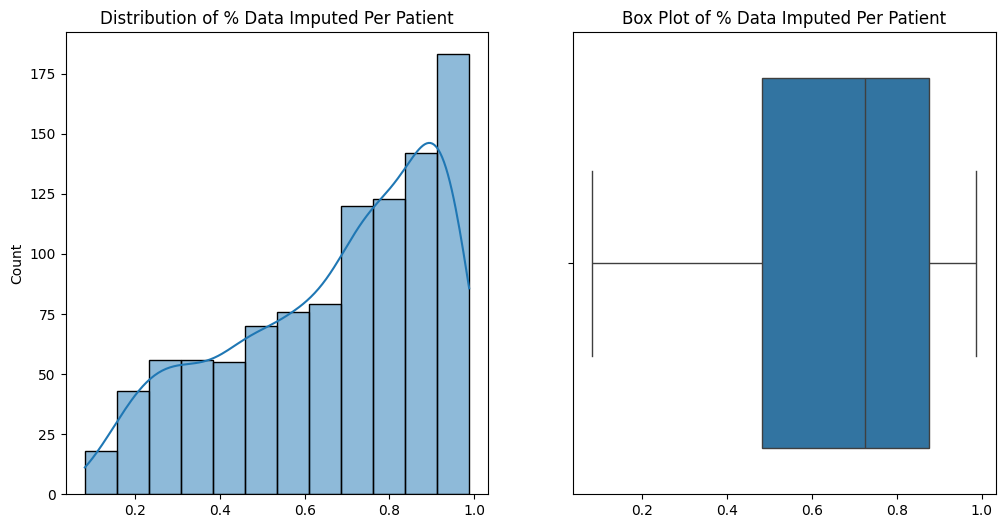
**
